# Mobilizing human capital in information technology projects: Interactions, negotiations, and actions of distributed actors

**DOI:** 10.1371/journal.pone.0325802

**Published:** 2025-06-13

**Authors:** Shrihari Suresh Sohani, Manjari Singh, Biju Varkkey

**Affiliations:** 1 Indian Institute of Management Indore, Indore, Madhya Pradesh, India; 2 Indian Institute of Management Ahmedabad, Vastrapur, Gujarat, India; Wroclaw University of Economics and Business: Uniwersytet Ekonomiczny we Wroclawiu, POLAND

## Abstract

The study explores the interaction of two cross-functional distributed actors (HR and project managers), their actions, and how they utilize existing practices to mobilize human capital in projects. The study, located at the intersection of practitioners and praxis, explores how the flow of different HR activities is constructed during project execution. Based on extensive field-based qualitative research and anchored on the micro foundations’ lens, we find that paired distributed actors (project manager and HR manager) engage in two mutually exclusive strategizing practices (human resource procedurally embedded and human resource interactive and mutual) for shaping the activity flow of HRM implementation. This finding is critical because it explains how the tension between the paired distributed actors is handled during project execution in project-based firms.

## Introduction

Strategizing is defined as “actions, interactions, and negotiations of multiple actors and the situated practices that they draw upon in accomplishing that activity” [1], p. 8]. When understood in the context of managing human resources in projects, it has been described as a process that “combines search, negotiation, confrontation, decision, and commitment (deal-making) activities” [2, p. 447]. While mainstream human resource management (HRM) research has primarily focused on three levels—the firm, unit/group, and individual—there is a growing need to examine HRM at the project level, which functions as a “temporary organization” [3]. This is particularly relevant in project-based firms, where HR and project managers must collaborate despite their distinct roles and priorities.

Although prior research has acknowledged interactions between HR and project managers, there is limited understanding of how these interactions impact project outcomes [4–6]. Existing studies have not thoroughly explored whether these interactions lead to unexpected challenges, inefficiencies, or conflicts during project implementation. For instance, tensions may arise due to differing objectives—HR managers focus on long-term workforce planning, whereas project managers prioritize immediate project deliverables [7]. Understanding how these differences shape HR strategy implementation in project-based settings is critical and hence is the purpose of our article.

Recent studies [8] have started examining shared responsibility in HRM implementation. However, current literature lacks clarity on the mechanisms that enable coordination between distributed actors across project and HR functions. Specifically, it remains unclear how HR and project managers align their efforts to implement HR strategies effectively. What specific mechanisms facilitate successful collaboration? What challenges hinder this coordination? [9]). This study aims to address these gaps by analyzing how distributed actors across these two functions implement HR strategies and construct activity flows for operationalizing HRM.

Our research is particularly relevant to the information technology (IT) sector, where project-based work is common. IT projects require both the exploration of new skills and the efficient utilization of existing capabilities. These projects operate in fast-paced, innovation-driven environments, making workforce agility and strategic HRM essential. Despite these unique challenges, the role of HR in IT project management remains underexplored. Do IT projects face distinct HRM-related obstacles compared to other industries? How do HR and project managers navigate talent acquisition, resource allocation, and employee retention in this context?

We investigate project-based information technology firms, where both HR and project managers must collaborate to ensure project success. IT firms rely on structured governance mechanisms [8] to coordinate resources across projects, yet little is known about how HR and project managers navigate these structures to achieve their respective goals. Given the time-sensitive nature of IT projects, distributed actors across project management and HR functions must align their strategies to ensure success [10,11]. This study contributes to the literature by providing empirical insights into these interactions, identifying challenges, and proposing mechanisms that facilitate effective collaboration.

## Theoretical foundation and research objectives

### Micro foundational thinking in PBO and HRM

Micro-foundational thinking is a recent phenomenon, with most empirical work arising from 2010 onwards in reputed journals [12,13]. In the field of project management, foundation thinking has been utilized to examine the interaction between contracting parties [14] and acts of “value-enhancing integration mechanisms” [15]. Pemsel et al. [16] utilize the micro foundational approach to understand knowledge governance in project-based firms. In a recent study, Bredillet, Tywoniak, and Totonac [17] utilized the micro foundation lens to understand the routine perspective as a unit of analysis in examining the co-evolution of the project management office and product portfolio management as an organizational capability. Eriksson and Kadefors [18] expressly point out the cognitive ability of project managers in large projects to develop routines specific to the project and the environment in which these projects operate. Such cognitive abilities are said to be the micro-foundations of the developed organizational routines. Therefore, Project management literature has started giving importance to the micro foundation lens in understanding emergent phenomena, especially when distributed actors construct it socially.

Specifically, HRM research has also supported micro-foundational thinking in understanding team-level agility [19]. Recent research is finding value and obtaining encouraging results when issues in HRM are being observed at the micro level, utilizing the micro foundational view [20,21].

As per the micro foundational lens, the organizational analysis should be concerned with how individual-level actions and factors aggregate to influence macro-level results (10). Social interactions and interdependencies among different actors in an organizational setting can lead to either incrementally positive or negative outcomes. Incrementally positive outcomes, mostly labeled ‘synergies,’ can effectively enact organizational strategies at operational levels. We, therefore, utilize the micro foundation’s view in our study, as the micro foundations’ lens allows us to “*put the locus of human capital resources at the level of interpersonal interactions*” [22]. It also allows us to understand *“How can such a firm use groups and teams to acquire, develop, and then mobilize and deploy the new human capital resources? How does one coordinate or revise existing teams to make this transformation? “*[22, p. 366–367]*.,* thus bringing forth the role of distributed actors.

Micro-foundational theory allows to bridge the gap between HR strategies and project execution by focusing on individual behaviours, interactions, and motivations. Most prominent micro-foundational studies have focused on the social factors influencing individual actions, which are then aggregated to produce macro-level social outcomes [23]. However, the complexity of social interactions and how individual actions shape strategic outcomes in projects remains under-explored. Applying micro-foundational thinking to understand the strategizing actions of distributed actors in projects offers a valuable approach for addressing this gap.

The theoretical foundation outlined above forms the basis for this research objective, which aims to explore the interaction between two cross-functional, distributed actors—HR managers and project managers. The study will examine their actions and how they leverage existing practices to mobilize human capital in project settings. The literature acknowledges that there must be a presence of value-enhancing integration mechanisms and practices for social construction. By utilizing these mechanisms and practices, an activity flow is created [24]. The structure of activity flow is contingent on the actor’s cognitive ability within the firm and macro context [25–27]. Therefore, HRM strategizing is a complex, dynamic process influenced by social construction, distributed decision-making, cognitive factors, and contextual conditions. Successful construction and implementation of HRM activity flow requires continuous adaptation, interaction among actors, and integration with broader organizational and institutional frameworks. Our study explores previously unexplored attributes of value-enhancing practices critical for building the activity flow. Such detailed elaboration of HRM implementation activity flow will extend the literature on strategy implementation within projects.

**Fig 1:**
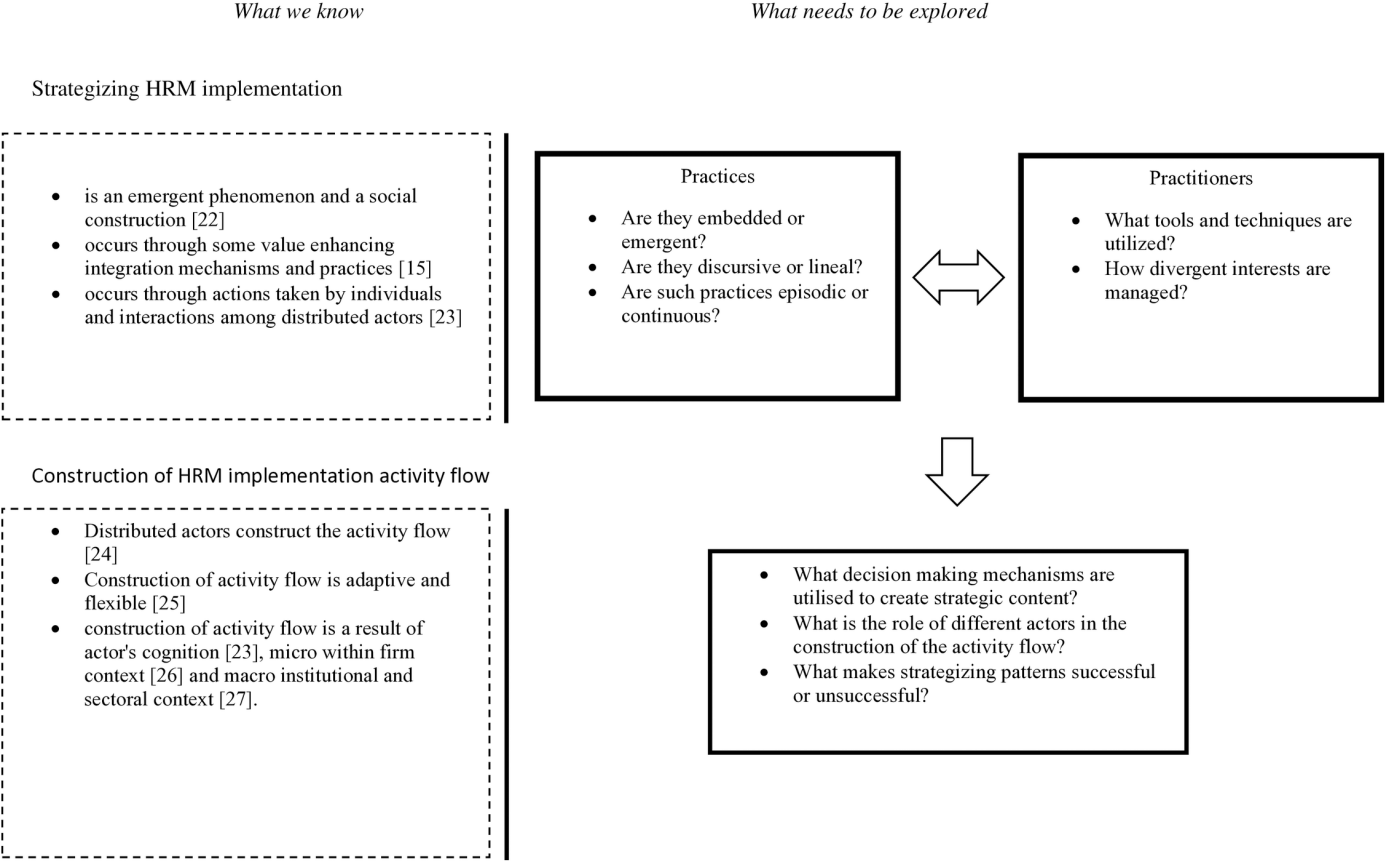
Conceptual framework and existing gap.

Fig 1 presents the conceptual framework of our study.

## Methodology

The study draws on extensive field-based research investigating how distributed actors implement HRM strategies. In this study, we utilize the inductive Gioia method. The foundation of the method lies in a rigorous inductive approach. Through this approach, we focus on concept development that allows us to specify “ *a more general, less well-specified notion capturing qualities that describe or explain a phenomenon of theoretical interest*” [28]. This method is beneficial when the process of the phenomena is under exploration. Focus on processes brings forth the appreciation of social construction [29,30]. As our study deals with understanding the flow of different HR activities, the Gioia method is best suited as it allows inductive concept development. By applying an inductive, qualitative approach (Gioia method) and focusing on distributed actors in project-based settings, our methodology effectively captures the social, cognitive, and contextual dimensions of HRM strategizing. It enables a rich exploration of how HRM activity flow emerges, adapts, and integrates within organizations, aligning perfectly with our conceptual framework. The Gioia method allows us to track how HR and project managers co-evolve HRM practices. Such method provides deep insights into emergent value enhancing strategizing processes.

We followed the procedure of purposeful sampling suggested by Lincoln and Guba [31]. Our sample comprised HR and project managers. Including project leadership as a distributed actor was essential, as they are critical in handling either exploratory, exploitative, or both sub-projects. Hence, purposeful sampling of such distributed actors was essential to addressing the crucial research questions of our study. Senior executives, purposefully selected, brought the management insight into the study that enabled us to understand their perception of the implementation of HRM strategy. The IRB approval was taken from the research and publications office of the institute of the first author. The authors collected the Data from 10th March 2024–25th June 2024 with informed consent from the participants. The participants were explained the purpose of the study and explicitly asked if they wanted to go forth with participating in the study. The authors recorded the verbal consent in the research notes.

All information technology firms that were approached for data collection were carefully selected. To understand HRM strategy implementation as an emergent activity, it was essential to explore project-based firms that earned considerable revenue from the execution of fixed-priced projects. The sample comprised 34 HR and project managers (with experience ranging from five to 25 years) from five information technology firms, with more than 50 percent of their revenue from fixed-priced projects. Table 1 provides a snapshot of the sample characteristics.

**Table 1:** Sample characteristics.

Sample Characteristics		Number of Participants
Function	HR function	12
	Project function	22
Experience (HR Function)	≤ 15 years	5
	> 16 years	7
Experience (Project Function)	≤ 15 years	13
	> 16 years	9
Firm	Firm 1	10
	Firm 2	7
	Firm 3	7
	Firm 4	4
	Firm 5	6

### Data collection

A full list of data sources, techniques of data collection, the data collected, and salient points of data collection are presented in Table 2.

**Table 2:** Source, collection techniques and salient points of data collection.

Data source	Human resource function	Project management function
*Interviews*: semi-structured, notes taken during interview, taped when allowed	12	22
*Participant meeting observations*: Archival status reports submitted and minutes of meetings	1) Group-level resource allocation meetings: 12 2) Project sourcing meetings: 9 3) Appraisal negotiations meetings: 4 4) Monthly recruitment calls: 15	
*Non-participant meeting observations*: Archival status reports submitted and minutes of meetings	1) Business unit-level attrition meetings: 4 2) Business unit-level contractual employees meetings: 2	
*Other documents*	1) Project plans for executed projects: 30	

### Salient points

- *Interviews*
  - Multiple in-depth, semi-structured interviews lasting about 60 to 90 minutes
  - Each business unit involved similar participants and had standardised or similar titles.
  - Collected various artefacts of strategizing that were utilised by the HR and project managers.
- *Triangulation*
  - Triangulation was carried out through the incorporation of:
    - Multiple interviews with respondents,
    - Observations during meetings,
    - Archival data of prior and existing projects, and
    - Analysis of artefacts utilised for strategizing.
- *Interview guide*
  - Three primary dimensions:
    - Interests of distributed actors
    - Mechanisms of collectivisation
    - Generation of the output of goal-directed actions

The interview guiding questions were carefully designed to cover multiple dimensions of project execution, HR challenges, and collective action mechanisms in organizations. The selection was made in consultation with two academicians and validated by four project managers and four HR managers to ensure relevance and applicability.

Table 3 provides the guiding questions and sources that we accessed for the same. The first set of questions provide foundational insights into the respondents’ experiences, roles, and work structures. The second set of questions explore the interest of distributed actors. These questions intend to examine the HR challenges in handling innovation-oriented and routine work modules, as well as interactions with HR and technical managers. The third of questions focus on retaining skilled employees and recruitment variations to provide insight into workforce planning. The last set of question explore mechanisms for collective action. The guiding questions together address project execution, HR concerns, role-based experiences, and organizational mechanisms.

**Table 3:** Interview guiding questions.

#	Focus	Sample guiding questions	Source
GQ#1	General	‘Please tell us briefly about your past projects and current project.’	Practitioners
GQ#2	General	‘Please tell us briefly about how work was structured in the projects that are executed in your organisation.’	[7]
GQ#3	General	‘How difficult is it to execute different type of work modules?’	[7]
GQ#4	General	‘Tell us about your current role.’	Practitioners
GQ#5	Interests of distributed actors	‘What exact HR challenges do you face while executing a complex and innovation-oriented work module?’	Practitioners
GQ#6	Interests of distributed actors	‘What are the usual points of agreement and disagreement with the HR manager?’	[4]
GQ#7	Interests of distributed actors	‘What exact HR challenges do you face while executing a typical work module that is low on innovation but an important part of the project?’	[24]
GQ#8	Interests of distributed actors	‘What issues do you face while dealing with technical managers?’	[4]
GQ#9	Generated output	‘What efforts do you make in order to retain the desired employee?’	Practitioners
GQ#10	Generated output	‘Do you recruit differently based on the nature of the work module?’	[8]
GQ#11	Generated output	‘How do you retain important employees?’	Practitioners
GQ#12	Mechanisms for collective action	‘How does a project manager raise a request for the HR function?’	Practitioners

### Data analysis

#### Coding of data.

The data were coded from original interview documents and observation sheets. Throughout the analysis, we iteratively refined the coding process to address anomalies and ensure consistency. Cross-coding was employed to validate responses and assess contributions to strategic activity. To enhance the trustworthiness of the data, we utilized triangulation, continuous engagement, and participant confirmation [31]. Thus, by integrating various data sources, the study mitigated potential biases and strengthened its empirical foundation.

We initially pursued an inductive analysis approach [26], followed by the constant comparison technique [32]. Inductive analysis was chosen because it allows patterns, themes, and categories to emerge naturally from the data, rather than imposing predefined frameworks. This approach ensured that our findings were grounded in the participants’ experiences and contextual realities. By working from specific observations to broader generalizations, we were able to capture nuanced insights and develop a theory that accurately reflects the complexity of HRM implementation. The combination of inductive analysis and constant comparison ensured a rigorous and systematic examination of the data. The analysis began by identifying fundamental concepts, which were grouped into categories using open coding. First-order categories, based on participants’ language, were then subjected to axial coding to reveal emerging themes. Finally, these themes were synthesized into aggregate dimensions to construct a comprehensive understanding.

### Analytic process and themes

Data collection for interviews began with broad questions exploring the roles of HR and project managers in fulfilling their responsibilities. As interviews progressed, questions were refined to address critical research problems in the qualitative study. Data were first coded based on activities carried out by the two actors. The primary focus of each actor was on the acquisition, utilization, or retention of human resources. We drew upon the preliminary interviews with respondents, data from the minutes of previous meetings, and artifacts such as human resource acquisition sheets to draw the correct phase-wise narrative of each activity [33]. The derived narratives were then profoundly analyzed to understand the influence of HR and project managers in operationalizing the HRM implementation strategy. The two most essential practices consistently evident were the utilization of formal HR procedurally embedded practices and the practices that involved interaction and face-to-face episodes.

After identifying the two most critical practices, we systematically coded all essential data related to rules and procedural mechanisms. This included aspects such as team composition control, employee appraisal and performance management, and HR budget finalization for the upcoming quarters. Through interviews and project artifacts, we examined how procedures were applied in activity construction. We then analyzed the relationship between the collected data and the corresponding activities, iterating our findings against existing literature to validate the emerging concepts.

Through this analytical process, we conceptualized HR managers’ use of formal procedures to shape implementation strategies as “HR procedurally embedded practices.” We coined this term to reflect their administrative foundation, which provided transparency and structural legitimacy. This procedural approach allowed HR managers to integrate activities within an established framework of rules and protocols, enabling them to implement strategies without direct intervention or oversight from top management [34,35]. Additionally, it granted HR managers control over resource allocation for activity construction.

A similar methodology was applied to understand interactive practices among distributed actors. Face-to-face interactions, initiated by both HR and project managers, played a crucial role in influencing stakeholders’ participation in HRM implementation strategies. These episodic interpersonal exchanges enabled HR and project managers to communicate their expectations and interpretations of the necessary strategic actions. Consequently, these interactions were often characterized by power asymmetries and played a dynamic role in shaping contributions to activity creation [36].

Following the identification of these two practices, we assessed their impact on activity flow. Specifically, we mapped key HR processes—acquisition, utilization, and retention—to evaluate how these practices influenced strategic action. Activity system maps were developed to visualize the dynamics of activity construction and power shifts among distributed actors. Ultimately, this mapping process led to the identification of a realized strategy content for HRM implementation.

## Findings

HRM implementation in project-based information technology firms has emerged as a function of the interplay between two strategically distributed actors: project managers and HR managers. We observe that while staffing for exploratory work modules, the perspectives of the HR and project manager ran opposite to the concept of strategic alignment of goals of HR and project manager for project success. The firm’s policies bind HR managers and are at variance with project managers who may want deviation to handle specific project-level requests. In the subsequent section, our findings highlight the inherent tensions in implementing HR implementation strategies and how the two actors resolve the same.

### Tensions between paired distributed actors

Competing stakeholder pressures make an agreement between the two actors, the HR, and the project manager, difficult. HR managers are under the corporate mandate. The HR managers across firms stated they were under unrelenting pressure to keep the number of unassigned employees to any project lower than a number. This figure varied across firms but ranged from 10 percent on the lower end to 20 percent on the higher end. It is important to note that when an employee was on the bench or had not been allocated any project, he or she was under the supervisory control of the HR manager. However, an employee came under the supervisory control of the project manager after being allocated a project. The employees unassigned to projects were bucketed based on the number of years of experience. Each HR manager responsible for the deployment of employees had bucket-wise targets for the allocation of employees. Senior employees were the costliest entry on the firm’s wage bill; hence, there was little leeway provided to HR managers concerning the non-allocation of such employees to billable engagements. One of the HR managers commented:

*‘In every weekly meeting, we are pulled up for even the slightest deviation from the range allowed us.’ [Respondent 3, HR deployment manager]*

Due to such pressure, HR managers tried to push such senior resources onto project managers. However, such an attempt was resisted if the employee proposed for a position needed to have the required skills. HR managers were concerned about senior employees unwilling to be retooled and undergo training. As one of the HR managers commented:

*‘Seniors are apprehensive of coming out of their comfort zone of the technology they are proficient in. Such employees do not want to venture into new projects based on internal or external classroom training, as they think they would fail.’ [Respondent 11, HR Executive]*

On the same point, one of the project managers commented:

*‘HR managers sometimes become myopic and are only concerned with keeping their parameters in line. They often ask us to onboard a senior resource without knowledge of the project skill set. Such associates are cost heavy and need a lot of lead time to become billable.’ [Respondent 29, Project Manager]*

Another bone of contention was the disagreement on trained resources and experienced resources. One project leader said:

*“The client does not desire trained resources on the project, and they want experienced professionals to be deployed. The corporate sourcing team tries to push trained new joiners to join the team. [Respondent 15, Project Manager]*

### Agreement between paired distributed actors

Although we observed a prevalence of differing agendas during the interactions between HR and project managers, we also found common threads that bound them together. The strands of this thread were predominantly the common objectives and values they shared. The constant focus on customer delight was a common purpose that compelled the distributed actors to look for solutions posed by paradoxical demands. Both the actors realized that constant customer satisfaction was necessary for business sustenance and for developing traction with existing and new customers. As one of the HR managers stated:

*‘We have to ensure that the project leadership is successful in their project execution. If projects are unsuccessful, the business unit will suffer from revenue pressures, which is not good for us.’ [Respondent 11, HR Business Leader]*

Another HR manager expressed:

*‘We are not a government department that is functioning in its silo and is immune to overall performance. We have to add value to the organization by contributing to the success of the engagements.’ [Respondent 25, HR Manager]*

The project managers seconded the agreement on shared objectives.

*‘Our HR managers do not have an easy job. They are under pressure from the head office to maintain adherence to basic workforce parameters, but they also ensure that projects do not suffer and that their demands are met. It takes much discussion, but they care for our genuine demands.’ [Respondent 15, Project Manager]*

An organization’s shared value system also drove congruence among the distributed actors. Both actors duly recognized that gender diversity and the inclusion of team members from different backgrounds and nationalities added to the success of the projects. One of the project managers commented:

*‘We have experienced that women bring a lot of balance and stability to the team. Along with the HR manager, we ensure a minimum 30 percent representation by women in our teams.’ [Respondent 32, Project Manager]*

We also find that organizational values, such as accountability and transparency, are critical in building consensus. Automation of actions also enhanced the transparency of the interactions between HR and the project managers. When a project leader raised a resource requirement in the system, its ownership was assigned to the required HR managers, and the status of each request was visible to all concerned. This led to accountability that compelled action and subsequently delivered results.

One of the HR managers stated:

*‘We all know who is responsible. Every request is allocated against a name, showing the days it has been pending, with no action. Our senior managers track the age of the requests and issues, and we have to attend to them and resolve them within the given service-level agreement (SLA).’ [Respondent 28, HR Manager]*

Similarly, a project manager was responsible for the request’s initiation date, and any acceptance was recorded in the system. To ensure transparency and accountability, the HR manager only acted on requests once they were raised in the firm’s human resource information system. Moreover, HR managers had access to utilized cost budgets and other project financials. We further observed that these points of agreement between the distributed actors allowed them to resolve the divergence among them and assist in the speedy implementation of the HRM strategy for engagement.

### Resolving tension between distributed actors: Role of strategizing practices

A significant component of the praxis of HRM implementation is the utilization of two practices. One provides a means for formalization, and the other allows for episodic interaction. This section examines the practices that influence the shaping of HRM implementation in the presence of tensions between distributed actors. These two practices can be designated *HR procedurally embedded (HRPE) strategizing* and *HR interactive and mutual (HRIM) strategizing*. In the subsequent subsections, we discuss the two strategies in detail and examine the implications of the two practices in shaping strategy.

### HR procedurally embedded strategizing

The primary role of HR’s procedurally embedded strategy is to embed and exercise’ diagnostic control’ over strategy (33). Such strategizing provides the necessary ‘structural legitimacy’ to multiple HR activities. This structural legitimacy is required for the necessary continuance of the activity. One such activity is budget finalization for the quarter. The HR manager oversaw the aggregation of the future workforce and their skill and level of expertise requirements. This enabled them to have a forward view of the requirements, allowing them some bandwidth for planning such demands. One of the module leaders commented:

*‘We have to contact our sales team and anticipate our workforce requirement for three months in advance. HR department circulates an Excel tool that has columns for multiple details. We fill it up for every account. This is an important and compulsory activity.’ [Respondent 16, Module Leader]*

Another similar activity was monitoring the performance management of team members and understanding the requirements associated with the training of associates. The HR managers also had procedural practices that ensured the desired ratio of experienced to non-experienced associates in the projects, as the project approval committee approved.

*‘The HR manager has the right to see the cost budget of the project and check if the project team is complying with the parameters that were approved for the project. Any deviation concerning the workforce, such as hiring senior resources instead of an approved junior position, needs approval from our side. Once the project ends, we must find another assignment for the hired employee.’ [Respondent 18, Senior recruiter]*

HR procedural strategizing involved the creation of requirement plans, associated budgets, and indicators for gauging adherence to the approved HR parameters for engagement. The requirement plans and related budgets indicated structural embedding. These allow for the sourcing of the activity and the necessary coordination for the implementation of the HR strategy. We observed that HR procedurally embedded strategizing provided an activity stream, which was encapsulated within a set of repetitive practices that would formalize ends and indicate responsibility for the stepwise execution of activities that were aimed towards the attainment of the indicated ends [37],formal documentation of activities allowed attachment of resources, specified ends, and associated responsibilities.

Apart from structural legitimacy, the indicators for adherence to HR parameters pointed to diagnostic control. Such a control mechanism provided necessary feedback and helped to conduct course corrections to ensure sticking to the agreed-upon path for resolving tensions while implementing the HRM strategy. Such control enabled course correction without excessive management intervention, as activities were rule-based [38]. Performance indicators as controls shaped the actions of distributed actors—project and HR managers—through associated benefits and sanctions. Therefore, HR procedurally embedded HR strategizing did not require much direct intervention from the distributed actors associated with the work. However, the benefits and sanctions associated with the work assisted in embedding desirable actions and weeding unwanted actions.

### HR Interactive and Mutual Strategizing

HR interactive and mutual strategizing allude to face-to-face interactions among HR and project managers to direct the flow of strategy for implementing HRM strategy. Such strategizing involves direct communication in each other’s presence; hence, it is one of the most potent resources because it involves immediate focus from interacting distributed actors [38]. This strategizing is not self-perpetuating in nature but dynamic. It allows for creating meaning and normative controls to lend interpretative validity to activity. The dynamic nature allows for the framing and reframing strategies so that these strategies continuously remain aligned with the end goal.

Interactive and mutual strategizing is critical in countering resistance because discussions in the presence of each other reduce time-related to the acceptance of the flow of activity. Concerning resolving tensions between distributed actors, interactive and mutual strategizing pertained to legitimizing interaction for acquiring and retaining human resources. One of the HR managers said:

*‘Our interactions start with the project managers once they have raised a request in the system for a resource. We get in touch with them and try to settle on a candidate. The discussions are usually “give and take,” where we take promises for future releases from their projects.’ [Respondent 30, executive, HR]*

Episodic interactions played a significant role in strategizing. The outcome of these episodic interactions was binding on the actors, providing necessary legitimacy and normative control over the event. For the retention of resources, the predominant HR activities, such as the distribution of rewards, special allowances, and appraisal ratings, also involved ad hoc meetings and discussions. One of the project managers said:

*‘Every year, we have a unit-level discussion with our HR managers for additional bandwidth, better appraisal ratings, and for employees engaged in difficult work modules.’ [Respondent 15, Project Manager]*

Another HR manager commented:

*‘Every quarter, we meet to discuss the cases for special project allowances. Such meetings are usually negotiations, where we provide special allowance to some employees. In return, we ask the project managers to allow such employees to train new members of different projects on their specialized skills.’ [Respondent 3, HR deployment manager]*

Interactive and mutual strategizing was critical to HRM implementation in a dynamic environment; interactions are vital to counter resistance to change. These strategies allowed the handling of any structural rigidity in the system. It provided the necessary dynamics required to avoid mean-end inversion over time.

**Fig 2:**
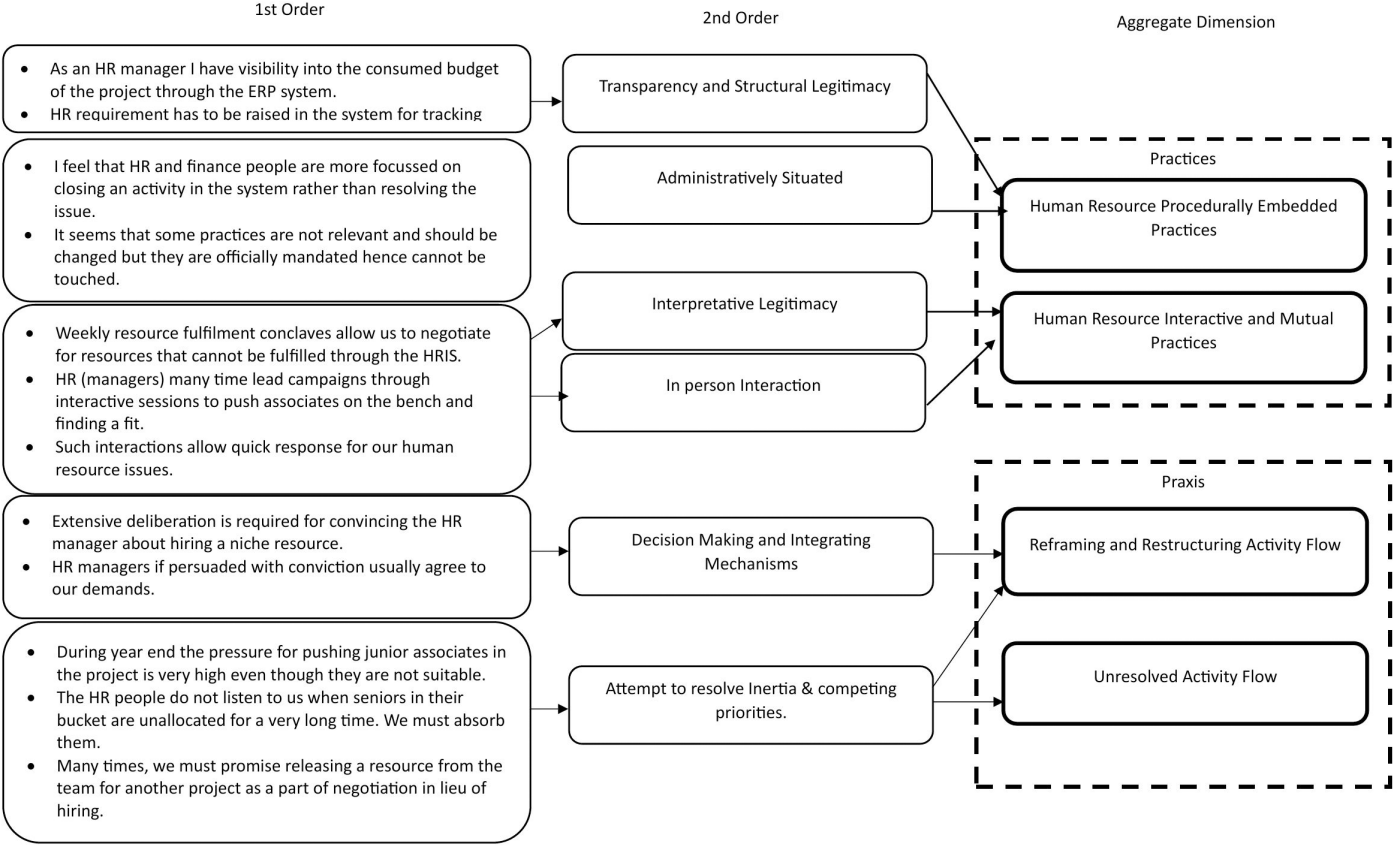
Data model.

We found that in project-based information technology firms, procedurally embedded and, interpretative and mutual strategizing balance each other for HRM implementation and avoiding strategic drift. HR procedurally embedded strategizing lent operational validity, and interpretative and mutual strategizing provided interpretative legitimacy. Fig 2 presents the data model of our study.

## Discussion and conclusion

The critical focus of microfoundations theory is understanding “how individual-level factors aggregate to the collective level” [39]. We address this call for explanation in our research. In this study, we extend the theory of micro-foundations in the context of projects by understanding the mechanism of creating collective action for HRM implementation at the project level.

Recent studies of institutional change and de-institutionalization have emphasized the agency perspective. This has primarily occurred regarding the configuration and reconfiguration of practices [40]. Firm-level studies such as those by Garud, Jain, and Kumaraswamy [41] point out the ability of actors to deconstruct existing practices and build new ones. This brings out the role of actors in adapting existing practices. Also, it is essential to understand under what conditions actors deem existing procedures outmoded or need modification. We study the collective players’ actions in acquiring, utilizing, and retaining resources to answer these questions. Our findings lead to the following proposition.

Proposition 1: Actors’ agency plays a key role in adapting and modifying existing HRM practices based on their perceptions of the appropriateness of these practices in the project context.

Another contribution of this study is the focus on strategizing practices and the importance of decision-making mechanisms used to construct strategic content. The literature points out the challenges posed by divergent interests among stakeholders. We extend the literature by exploring how such divergence requires frequent and high-intensity interactions to construct meaning continuously. Literature also points to praxis as a stream of activities and action patterns. Such activities and patterns connect the actions of different individuals or stakeholders [42,43]. We extend the literature by studying how creating a stream of activities contributes to successfully implementing HRM practices. The development of activity system maps in our study demonstrate how HR processes (acquisition, utilization, retention) influence strategic action. The visualization of power shifts among distributed actors provided tangible evidence of how HR and project managers manage tension shape HRM implementation.

We found that the quality of operationalization directly influenced the strength of the HRM system that was created. Thus, if the HRM process implementation is robust, it will create a consensus-driven, robust HR system. A strong HR system will lead to a healthy organizational climate that will result in the achievement of performance goals [44]. It has been noted that if the “process of HRM system is strong, a shared perception of the climate will emerge in organizational subunits” [45, p. 215]. We find that through the utilization of two strategizing practices—HRPE and HRI&M—the functionally distributed actors (HR and project managers) built a shared strong climate at the project level for the successful execution of HRM processes.

### Role of strategizing practices in building activity flow

Through strategizing practices and decision-making mechanisms, the two distributed actors (HR manager and project manager) built a shared interpretation of the situation they needed to address concerning acquisition, utilization, and retention. Our study focused on paired distributed actors in shaping strategies for implementing HRM. Our findings point to the inherent differences between HR and project managers. Further, for all the core activities, the distributed actors employ HR procedurally embedded strategizing to give structural legitimacy to such activities. HR interactive and mutual strategizing was useful in continuous adjustment and alignment for realizing desired strategic content. It provided interpretative legitimacy for activities taken together with procedurally embedded strategizing. This results in a framework that elucidates how the strategy for HR implementation as a flow of activity is formed over time.

Interactive and mutual strategizing are essential because they are complementary, and each type covers the weaknesses of the other. Our findings therefore lead us to propose that

Proposition 2: HR procedurally embedded strategizing and Interactive strategizing and mutual strategizing complement each other, covering each other’s weaknesses and facilitating the creation of a continuous flow of activities that contribute to the effective implementation of HRM practices.

**Fig 3:**
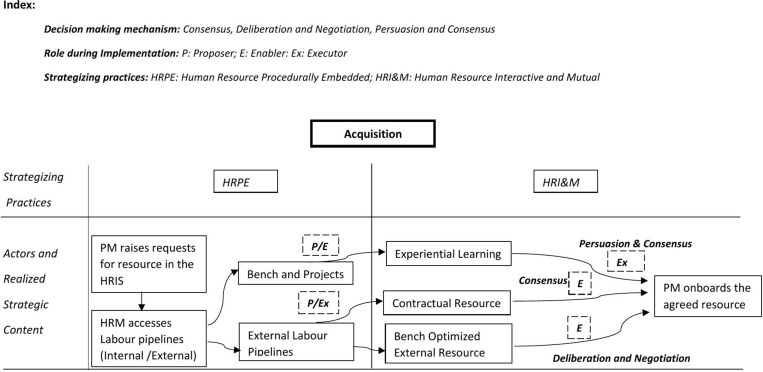
Practitioner -Praxis framework acquisition.

**Fig 4:**
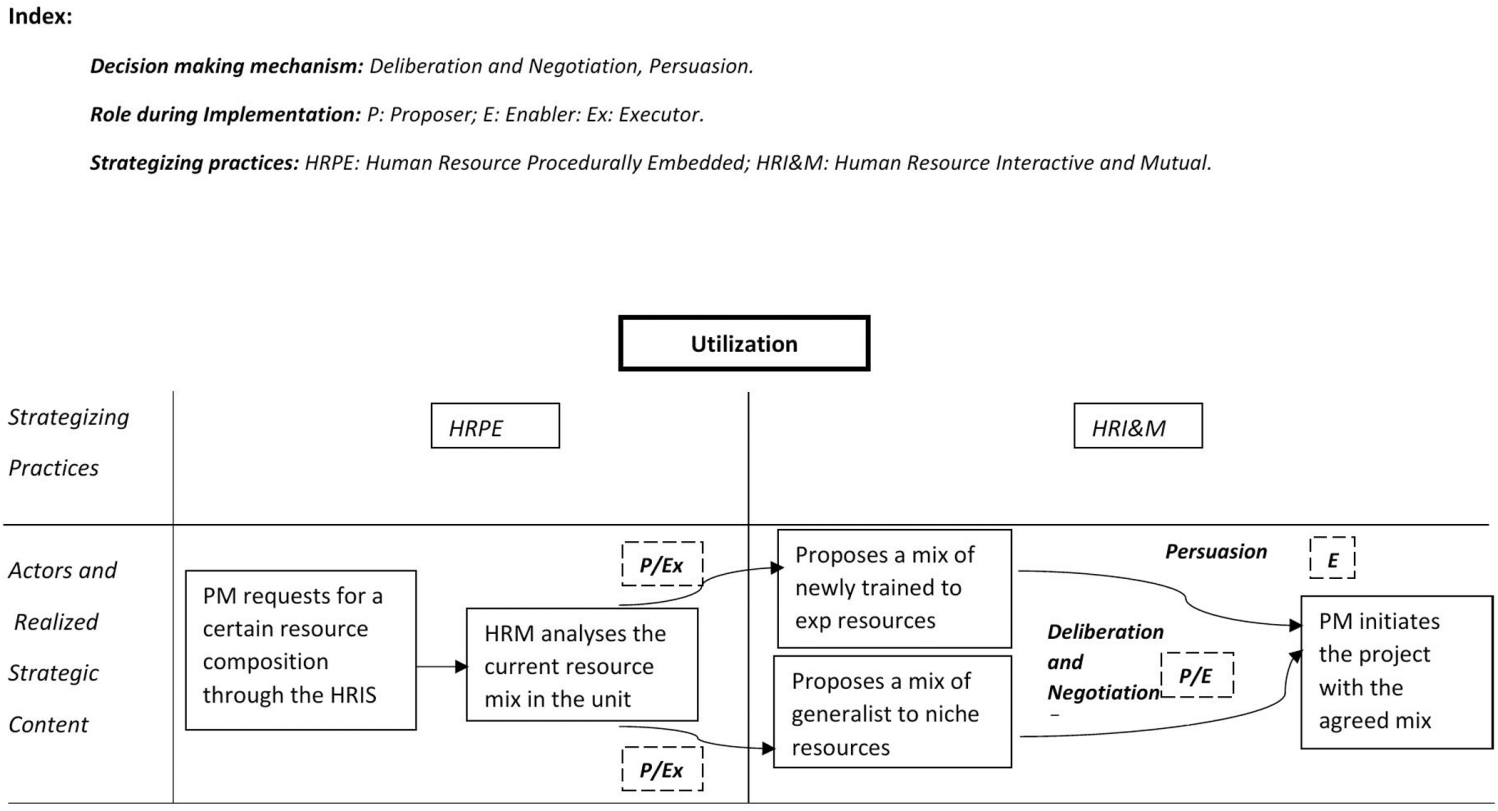
Practitioner -Praxis framework utilization.

**Fig 5:**
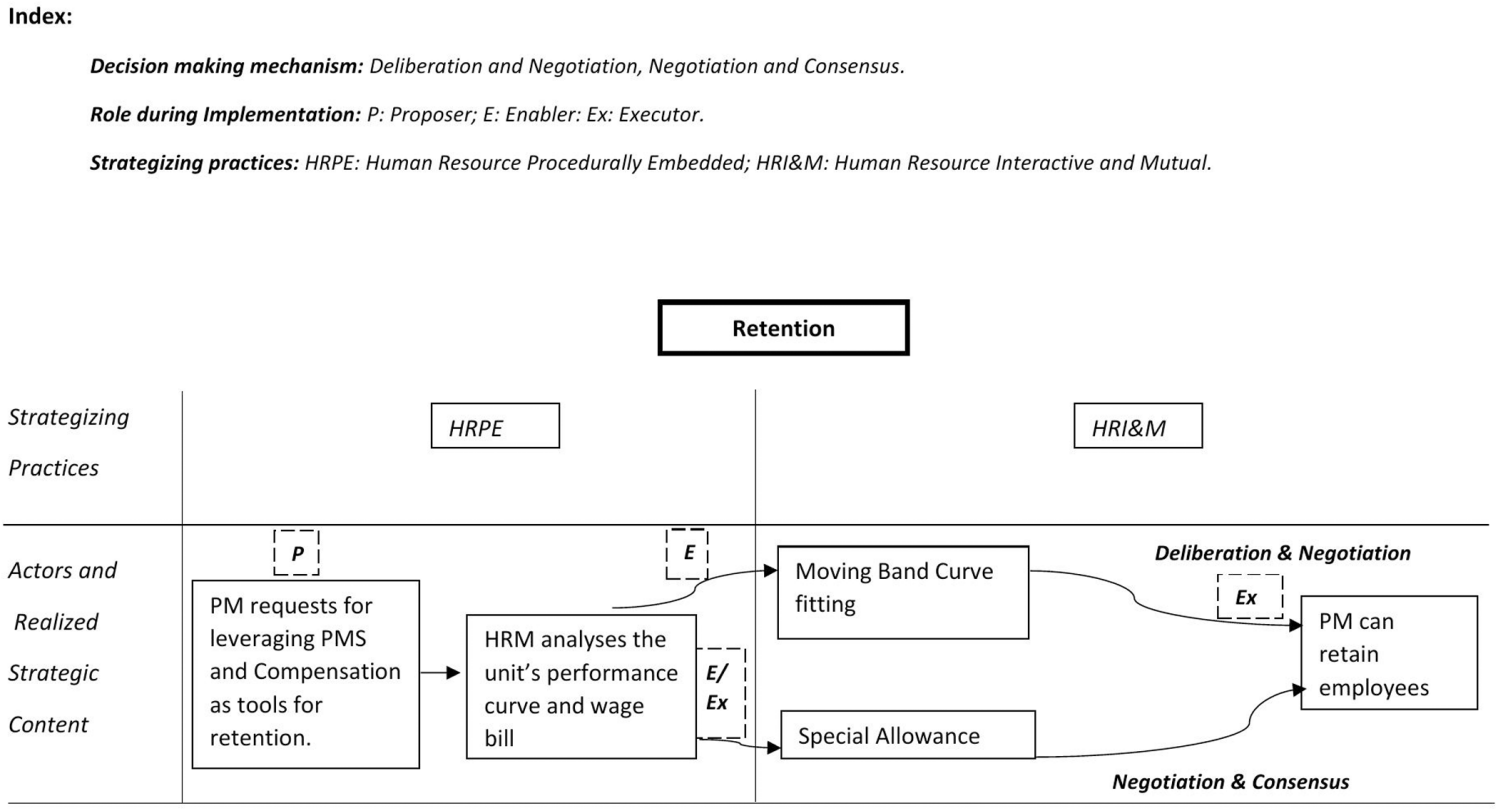
Practitioner -Praxis framework retention.

Different combinations of the two generate a flow of activity in different ways, thus creating a strong climate at the project level. Figs 3–5 details the practitioner-praxis framework for the acquisition, utilization, and retention processes. The figures also include the decision-making mechanisms for shaping activity flow.

The realized strategic content concerning the acquisition, utilization, and retention of resources is enumerated in Table 4. The table also presents the triangulation source for each strategic content. We can therefore conclude that

**Table 4:** Realised strategy content (Triangulation table).

#	Realised strategic content	Critical focus	Triangulation source
AR 1	Experiential Learning	Resource acquisition	Time-sheet Entries
AR 2	Contractual appointment-specialized contingent labour	Resource acquisition	Monthly recruitment calls & Business unit level contractual employees’ meetings. Direct observation Accompanying archival status reports Minutes of meetings
AR 3	Bench optimised sourcing	Efficiency of resource acquisition	Interaction between HR managers and HR providers E-mail Resource requests in the HR system
RU 1	Determination of resource mix	Resource utilisation	Interactions between HR and Project managers E-mail Minute of meetings
RR 1	Special allowance	Resource retention	Compensated individuals Discussion
RR 2	Moving band curve fitting	Resource retention	Unit-level appraisal meetings Direct observation

Proposition 3: A strong HRM system, formed through robust implementation processes, fosters a consensus-driven organizational climate, which positively influences the achievement of project performance goals.

The distributed agency provides a more realistic view of institutional processes as nondeterministic and of outcomes as contingent on the actions and reactions of distributed actors [38,46]. Distributed actors demonstrate contextualized knowledge, competence, and skill to extend or remodel practices. When tension in competing priorities exists, institutionalized practices are likely to fail to fulfill the mandate. The distributed actors then engage in interactive and mutual strategizing to attain the desired outcomes. Jarzabkowski [35] states that micro-communities of players pursuing divergent strategic goals are fertile ground for developing novel practices. Our practitioner praxis framework highlights the development of innovative processes utilizing multiple decision-making mechanisms by distributed actors.

Project managers and human resource managers diverge from established institutions in a particular environment to achieve the project goal. Our research contributes to the field of organization theory by reviving its mandate to address the role of individual actors in the more extensive social system by attempting to comprehend how they contribute to changing the organizational environment in which they are embedded despite institutional pressures toward stability [47].

Our study led us to articulate the mechanisms and the content of strategic action for implementing HRM to handle paradoxical requirements in projects. Distributed actors, driven by disparate goals, must collaborate uniquely to realize HRM strategy. While our findings offer valuable insights, they are specifically drawn from the IT sector, where projectized ways of working, distributed teams, and innovation-driven environments create unique HRM challenges. Our study is highly relevant to IT project management but may not fully apply to industries with different work structures, such as manufacturing.

Given this sector-specific focus, future research should explore how these interaction mechanisms function in non-IT environments, particularly in mechanistic organizations with larger and more hierarchical structures. For instance, manufacturing firms often involve more numerous and rigidly structured actors, leading to challenges such as communication breakdowns and resistance to collaboration. Investigating how HR and project managers in such environments engage in strategizing practices could offer additional insights.

Another promising direction for future research is the examination of failure cases in IT project-based HRM implementation. Retrospectively identifying and analyzing failed interactions between distributed actors is inherently difficult, yet it is crucial to understanding what factors lead to unsuccessful HRM strategy execution. Qualitative comparative analysis [48] offers a useful methodological approach to identifying which specific factors and combinations contribute to failure versus success.

By focusing on IT firms, our study contributes to organizational theory by reviving its core mandate—understanding how individual actors influence and reshape the broader organizational environment despite institutional pressures for stability [47]. As IT firms continue to evolve, a deeper understanding of distributed agency, strategizing practices, and HRM implementation mechanisms will remain critical for sustaining effective project execution.

## Supporting information

S1 Table Sample quotes. (DOCX)
