# Supplementary material for: Mobilizing human capital in information technology projects: Interactions, negotiations, and actions of distributed actors
Source: PLoS One. 2025 Jun 13;20(6):e0325802. doi: 10.1371/journal.pone.0325802 (PMC12165412; doi:10.1371/journal.pone.0325802)
Supplement: S1 Table — (DOCX) [file pone.0325802.s001.docx]

**S1 Table: Sample quotes**

1. “Through the ERP system, I can track the project's consumed budget in real time. “
2. “We will need to raise the HR requirement in the system. This helps us track the request properly and ensures smooth processing. Once it's logged, I can take it forward for approval and initiate the hiring process.”
3. "If you need insights into budget utilization, I can generate a report from the ERP system."
4. "Before approving new hiring requests, I always check the consumed budget in the ERP system to ensure alignment with financial plans."
5. "To streamline hiring, we require managers to submit HR requests in the system before proceeding."
6. "The system allows us to track all HR requests efficiently, ensuring there are no delays in recruitment."
7. "Project processes should serve project leaders, not the other way around—but too often, the system dictates our actions instead of logic."
8. "We continue certain practices not because they add value, but because they’ve been in the organization for a very long time”
9. "Engaging sometimes heated discussions in our resource meetings help us identify and deploy the right people quickly."
10. "Securing approval for a niche hire often requires thorough justification and multiple discussions with the HR manager."
11. “The strength of case has to be solid to convince HR to hire a high prized specialised resource”
12. “The HR mangers are usually cautious but when they see the value they become our allies”
13. "HR prioritizes placing unallocated juniors into projects, this is usually not the best thing to do as they take a lot of time to come up to speed. The customer becomes unhappy"
14. “Hr is always trying to balance its resource pool. Project requirements many times take a back seat”
15. “Some times workforce management becomes more a case of give and take with the HR managers”
